# Supplementary figures and images for: Abundance, size, and survival of recruits of the reef coral Pocillopora acuta under ocean warming and acidification
Source: PLoS One. 2020 Feb 4;15(2):e0228168. doi: 10.1371/journal.pone.0228168 (PMC6999881; doi:10.1371/journal.pone.0228168)

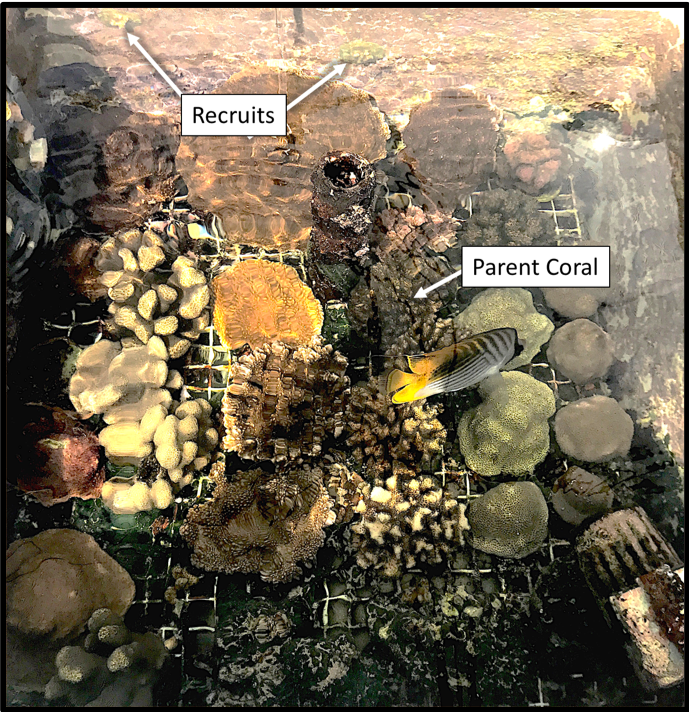

Supplement: S1 Fig — (TIFF) [file pone.0228168.s001.tiff]

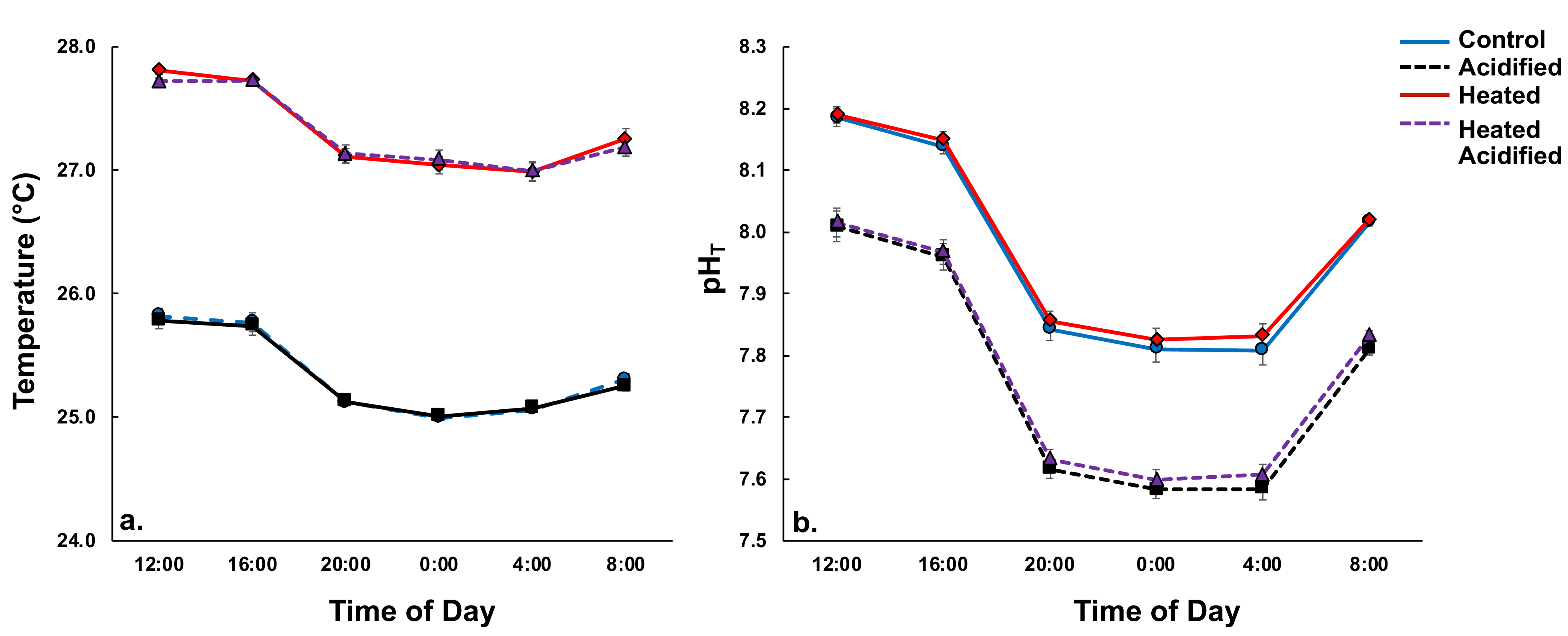

Supplement: S2 Fig — Diel sampling occurred quarterly throughout experiment. Data shown here is from 27–28 June 2017. (TIFF) [file pone.0228168.s002.tiff]
