# Supplementary material for: Abundance, size, and survival of recruits of the reef coral Pocillopora acuta under ocean warming and acidification
Source: PLoS One. 2020 Feb 4;15(2):e0228168. doi: 10.1371/journal.pone.0228168 (PMC6999881; doi:10.1371/journal.pone.0228168)
Supplement: Attachment — Submitted filename: Response to Reviewers.docx [file pone.0228168.s003.docx]

**We would like to thank the reviewers and the editor for these timely reviews. They have improved the manuscript tremendously. We have addressed all the concerns of the reviewers and included the responses here as well as tracked changes in the revised manuscript.**

**Significant improvements have been made to the manuscript structure including a literature update and more detail for the methods. We have also included more data as requested by Reviewer 2.**

**Reviewer 1**

The authors present a study that aim to quantify the abundance, size, and survival of the coral Pocillopora acuta that recruited inside experimental mesocosms under current and projected condition of ocean warming and acidification. Early life stages of corals are more sensitive to environmental change compared to their adult counterparts so the present manuscript face a relevant topic for the coral reefs ecology. I think the ms is worthy to be accepted for the publication on PONE after some major and minor revisions as follow.

**Authors’ Response:** Thank you for your comments on our manuscript. It has improved it significantly.

In my opinion the Discussion paragraph, need to be rearrange to better underline ms results. Generally, your results have been little debated while most of the text regards statements of other authors.

**Authors’ Response:** The discussion has been reorganized with more emphasize on the results and comparing to other studies.

The initial sentence (line 254-261) is redundant and more suitable for the Introduction. You should integrate it where you speak about the importance of the coral recruitment to predict future trajectories of coral reef ecosystems (line 57-64).

**Authors’ Response:** Lines 254-261 have been removed from the discussion and integrated into the Introduction as recommended by the reviewer.

Sometimes in the text there is no space between two consecutive words, check the document (line 42).

**Authors’ Response:** Thank you for catching this. We have reviewed the manuscript to ensure this has been corrected.

(Line 50-52) add this sentence or similar and references: “although, field studies revealed that even abiotic factors in the marine environment may interfere with the effect of ocean acidification on corals (Oprandi et al., 2019; Oporto-Guerrero et al., 2018; Januar et al., 2017) and biological feedbacks complicate such predictions (24)”.

Oprandi, A., Montefalcone, M., Morri, C., Benelli, F., & Bianchi, C. N. (2019). Water circulation, and not ocean acidification, affects coral recruitment and survival at shallow hydrothermal vents. Estuarine, Coastal and Shelf Science, 217, 158-164.
Oporto-Guerrero, T., Reyes-Bonilla, H., & Ladah, L. B. (2018). Presence of the reef-building coral, Porites panamensis, in a shallow hydrothermal field in the Gulf of California. Marine Biodiversity, 48(1), 703-708.
Januar, H. I., Zamani, N. P., Soedarma, D., Chasanah, E., & Wright, A. D. (2017). Tropical coral reef coral patterns in Indonesian shallow water areas close to underwater volcanic vents at Minahasa Seashore, and Mahengetang and Gunung Api Islands. Marine ecology, 38(2), e12415.

**Authors’ Response:** The following sentence has been added to the revised manuscript: “Calcification rates of reef building corals are estimated to decrease up to 15-20% by the end of the century due to ocean acidification alone (3, 14, 17-25) although, field studies have revealed that abiotic and biotic factors in the marine environment may mask the effect of ocean acidification on corals, obscuring its detection (26-28), and biological feedbacks may complicate such predictions (29).

**Reviewer 2**

Bahr et al reported the effect of warming and acidification on the recruits’ survival of the resistant coral Pocillopora sp. (recognised as P. acuta based on its morphology only) during a long-term experiment in aquaria.

This study could merit to be published likely as a note, therefore reducing citations, figures and likely some text, because although not innovative, it improves knowledge on the effect of environmental change on corals’ survival, specifically on their early life stage which it might be the major bottleneck.

**Authors’ Response:** Thank you for your comments and suggestions.

I have a number of questions and points the Authors need to clarify. My first, natural question to the Authors is: do you really planned this experiment or is it just an opportunistic way to publish a side paper from one main project? 40 tanks were used during a 22-months study to study recruits’ survival only of one brooder coral only! Settlement was observed on the tanks wall (no supports for settlement were provided)! The tank become the unit to be compared between treatments! There is no description of where the settlement happen (top, bottom, other supports, plastic, PVC, pump?). No description of the aquaria conditions (presence of CCA on the walls? Nude, clean aquaria?). How many colonies per tank? Their size? Did the Authors cleaned the aquaria? What about algae effect on the coral survival rate? How the Authors observed the recruits pigmentation? Visually I read, but in which way? Maybe using a camera underwater? Did the Authors ‘dive’ into the 70-L tanks? Is this method enough precise? Although the experiment started in March 2016 only recruits from June 2017 were reported. Why? These are the most prominent questions I had reading this ms.

**Authors’ Response:** This experiment was an experiment of opportunity that was part of a larger project. The following text has been added to the Introduction of the manuscript:

“Continuous flow-through experimental mesocosms were held at current and future projections of ocean warming and acidification to understand long-term impacts on coral reef communities. During this long-term experiment, we noticed high levels of recruitment within these experimental mesocosms. This provided a fortuitous opportunity to understand the impacts of ocean warming and acidification on coral recruitment (i.e., abundance, size, and survival) of Pocillopora acuta that recruited inside these flow-through experimental mesocosms.”

The following text has been added to the methods for clarification of experimental design:

“These experimental mesocosms were a part of a larger long-term project to understand ocean warming and acidification impacts on coral reef communities. To simulate a reef environment, each mesocosm included coral nubbins from all the major Hawaiian reef building species (*Montipora capitata, Montipora flabellata, Montipora patula, Pocillopora acuta, Pocillopora meandrina, Porites compressa, Porites evermanni,* and *Porites lobata*), yielding an initial coral cover of roughly 10%, a thin layer of carbonate reef sand, and pieces of reef rubble. The mesocosms were supplied with live, unfiltered seawater and a juvenile threadfin butterflyfish (*Chaetodon auriga*) and a juvenile convict tang (*Acanthurus triostegas*) were placed in each mesocosm to control algal provided the essential ecological functions of herbivory and predation in the mesocosms; therefore, mesocosms were not cleaned. Corals recruiting alongside the walls of the mesocosms were assessed for this study.”

We have also included a photograph of the inside of the mesocosms/tanks.

**Figure S1.** Photograph inside of experimental tank showing adult colonies and coral recruits.

Other points.
Because the difficult to distinguish among the different Pocillopora species, which could be found in sympatry, it should be critical to identify the colonies used in the experiment using classic and available mitochondrial markers.

**Authors’ Response:** Previous genomic work has shown that Pocillopora are genetically distinct using mtORF (Johnston et al., 2017) and these authors have shown that P. acuta is far more dominant in Hawai’i than P. damicornis – 24 out of 25 sampled individuals were P. acuta (Johnston et al., 2018).

The literature about OA is old!! Langdon, Kleypas, Gattuso, Erez, Smith made the story of OA, but this science has deeply changed and the story has improved during the last ten years. Please, cite one, two of these papers and report more recent ones. Specifically, the Authors forgot to mention (and to discuss!!) studies on the effect of OW and OA on the Pocillopora species. Because Pocillopora has become a model to study early life stages, some studies have been already published so far. Please, read at least Putnam and the last study by Bellworthy!

**Authors’ Response:** We have included updated references regarding OA including (Oprandi et al., 2019; Oporto-Guerrero et al., 2018; Januar et al., 2017).

I have a concern about the experimental conditions and the treatments. One measurement made at midday per week of the environmental parameters and averaged per month (and during the three study periods only) is not enough to describe the experimental environment. Any chance to have some more data?

**Authors’ Response:** We have provided the long term temperature data in Fig 1 in the manuscript and we have also added the long term pHT data. We have also included the diurnal data from our quarterly sampling in Figure S2. This figure has been added to the manuscript.

**Figure S2.** Diurnal fluctuations in temperature (a.) and pHT (b.) values in experimental treatments of Ambient (AT ACO2; blue solid line), Acidified (AT HCO2; black dotted line), Heated (HT ACO2; red solid line), and Heated Acidified (HT HCO2; purple dotted line). Diel sampling occurred quarterly throughout experiment. Data shown here is from 27-28 June 2017.

Finally, this study does not assess OA because pH is too close to ambient. Firstly, I was surprised to read pH 8.21 for the Ambient treatment which is quite high (i.e., pCO2 218 is a preindustrial value!). What’s more pH is in total scale value that is ca 0.1 less than measurements made in NBS scale. But more relevant to address my concern is that the High CO2 treatment have pHT 8.06-7.95 (654-478 pCO2). This is not what the Authors should test to assess the effect of OA and actually those values are in the normal range experienced even by front reef corals. Is there a mistake in the calculation? Honestly, I cannot accept it even if presented as “moderate acidification” in the last sentence of the ms.

**Authors’ Response:** The stated pH values (presented in Table 1 of the manuscript) are the mean Mid-day (12PM) values in a naturally diurnal fluctuating system. These mid-day values have among the highest pH values for all treatments due to high levels of photosynthesis. We also conducted diurnal sampling within all the tanks quarterly (~March, ~June, ~September, ~December) to capture the fluctuations in temperature and carbonate parameters. The figure below shows the pH values for all treatments by time of day. Here you can see the highest values of pH are at 12PM and the lowest in the at 4AM. This is also provided in the supplementary data.

Mean pHT values by treatment over a 24 hour cycle (June 2017): Ambient (AT ACO2; blue) Acidified (AT HCO2; black), Heated (HT ACO2; red) and Heated Acidified (HT HCO2; purple).

Carbonate parameters (pH_T_ and pCO_2_ (µatm)) by treatment for diurnal sampling that occurred in June 2017. Carbonate parameters for diurnal sampling will be provide in the supplementary data.
